# Values of First and Early Third Trimesters Serum Lipid Profile in the Prediction of Preeclampsia: A Cohort Study: Preeclampsia and Lipid Profiles

**DOI:** 10.31661/gmj.v11i.2395

**Published:** 2022-12-30

**Authors:** Azam Faraji, Behnaz Razavi, Marjan Zare

**Affiliations:** ^1^ Maternal-fetal Medicine Research Center, Department of Obstetrics and Gynecology, School of Medicine, Shiraz University of Medical Sciences, Shiraz, Iran; ^2^ Maternal-fetal Medicine Research Center, Shiraz University of Medical Sciences, Shiraz, Iran

**Keywords:** Preeclampsia, Lipids, Gestational Hypertension, Pregnancy

## Abstract

**Background:**

The global prevalence of hypertensive pregnancy disorders (HPDs) is5.2%-8.2%. Lipid profiles made up of triglycerides (TG), total cholesterol (TC), low-density lipoprotein-cholesterol (LDL), and high-density lipoprotein-cholesterol (HDL) could affect the arterial vessel wall leading to HPDs. Preeclampsia (PE), among the most severe maternal-fetal HPDs, has affected 0.2%-9.2% of all pregnancies. The current study aimed to investigate the role of lipid profiles in predicting PE in the first and early third trimesters of pregnancy.

**Materials and Methods:**

A large-scale prospective cohort study was conducted from early pregnancy onward in a normal population in the south of Iran. Fasting blood samples were examined for TG, TC, HDL, and LDL, as well as LDL/HDL ratio levels in the first and early third trimesters.

**Results:**

Of 486 pregnant women, 37 women developed HPDs, of which 20 (54%) developed PE. In the PE group, the levels of serum lipid profiles, including TG, TC, LDL, and HDL significantly raised with gestational age (P0.05). After adjusting for maternal age and body mass index, TG, TC, LDL, and LDL/HDL ratio levels were associated with a higher risk of PE (odds ratio [OR]=1.025, 1.035, 1.03, 2.08, and 1.026, 1.044, 1.03, 2.14, P0.001) regarding the first and early third trimesters, respectively. The optimum cut-off points for TG, TC, LDL, and LDL/HDL ratios predicting PE were estimated to be180.5 mg/dl, 197.5 mg/dl, 136 mg/dl, and 3.66 in the first, and 220 mg/dl, 204 mg/dl, 155.5 mg/dl, and 3.97 in the early third trimesters.

**Conclusion:**

Dyslipidemia during pregnancy may help predict PE development that can be sustained with lipid-lowering drugs.

## Introduction

Hypertensive pregnancy disorders (HPDs) have been recently raised with a global prevalence of 5.2%-8.2% [1]. Preeclampsia (PE) with a prevalence of 0.2%-9.2%, is the most notable complication of HPDs that is responsible for 10%-15% of maternal morbidity and mortality [2]. PE could induce severe acute complications in the fetus, infant, and child without proper treatment. Its most common complications are chronic hypertension (HTN), diabetes mellitus [3], fetal growth restriction, preterm birth, non-reassuring fetal status, intrapartum death, neonatal mortality and morbidity, and long-term cardiovascular complications in infants and children [4][5][6][7]. Frequently, clinicians follow a diagnosis of PE founded on the patient’s blood pressure and the presence of protein in the urine (proteinuria). Based on the American College of Obstetricians and Gynecologists (ACOG) guidelines, the PE judgment does not need to detect high proteinuria any longer [8]. Signals demonstrating limb malfunctions, especially liver and kidney, can happen with no signs of proteinuria; also, the amount of protein detected in the urine does not predict the severity of the disease. Also, the severity of the disease is attributed to the presence of blood pressure≥160/110, headache, visual disturbance, hematologic involvement, pulmonary edema, and fetal growth restriction [4][8]. Maternal hyperlipidemia is necessary for fetal growth and development; lipid profile levels increase meaningfully from the first to the second trimester of pregnancy [9][10]. In addition, there were higher odds of stillbirth, preterm birth, and fetal growth restriction with higher levels of triglycerides (TG), total cholesterol (TC), and low-density lipoprotein-cholesterol (LDL), in the first and second trimesters of pregnancy; however, lower odds were seen with a higher level of high-density lipoprotein-cholesterol (HDL) [9][10][11].

PE is caused by placental insufficiency and endothelial dysfunction associated with oxidative stress and dyslipidemia. Dyslipidemia in pregnant women is associated with oxidative stress, atherosis of the spiral arteries, and endothelial dysfunction [12][13]. Also, women with PE and high lipid levels during pregnancy are at risk of cardiovascular disease and HTN later in life [14][15]. Since lipid profile changes can predict, prevent, and sustain HTN with lipid-lowering drugs [16], the current large-scale prospective study aimed to investigate the diagnostic values of lipid profiles for predicting PE in the first and early third trimesters of pregnancy.

## Materials and Methods

### Study Population and Design

A cohort study was performed on 486 healthy pregnant women between October 2018 and February 2020. All participants were recruited from tertiary centers affiliated with Shiraz University of Medical Sciences as a referral focal point in southern Iran. The participants were followed prospectively from early pregnancy onward, and their HPDs status and lipid profile levels were recorded in the first and early third trimesters of pregnancy.

### Sample Size Calculation

Based on the information reported by Abalos *et al*. [2] giving the estimated proportion for PE=0.46, desired precision of estimate=0.05, at the confidence level (CI) of 95% using the following formula:


n = \frac{(p \times q) \cdot z^2_{\alpha/2}}{d^2}

In where z is the value from standard normal distribution corresponding to the CI of 95% (z=1.96), p is the expected proportion, q is one minus the expected proportion, d is desired precision (half-desired CI width), and 20% of attrition rate, the sample size was estimated to minimum 486.

### Inclusion and Exclusion Criteria

Inclusion criteria were singleton pregnancy up to 30 weeks of gestational age (GA) with a live fetus, clear GA, and natural conception. Exclusion criteria were diabetes mellitus type I and/or II, serious infections during early pregnancy, specific diets like gluten-free or casein-free diets, underlying diseases (e.g., heart, liver, and renal failures), chromosomal abnormalities, inherited metabolic diseases, rheumatologic or vascular diseases, hypercholesterolemia, thyroid disease, medication use for the regulation of glucose or cholesterol during the study enrollment, and chronic hypertensive disorders.

### Biochemical Analysis

Fasting blood samples were taken twice at first (0-14 GA) and early third (24-30 GA) trimesters of pregnancy for all participants. The samples were analyzed for TG, TC, LDL, and HDL level measurements. LDL and HDL measurements were done by homogeneous enzymatic colorimetric assays, while TG and TC were measured by cholesterol oxidase-phenol aminophenazone and glycerol3-phosphatase oxidase phenol aminophenazone methods. The lipid profiles were measured on automatic biochemical analyzer kits (BIOREX, Iran) specific for the detection of TC and TG, HDL, and LDL. LDL/HDL ratio was measured by the division of LDL by HDL. HPDs, gestational HTN, PE, severe and mild PE, and eclampsia were defined based on ACOG guidelines [8].

### Follow-Up and Data Collections

All participants completed a brief checklist about maternal age, height, weight, gravida, history of abortion, and GA at the time of entrance to the study and then were followed for their entire pregnancy period. Besides, a visit at 6-12 weeks postpartum was planned to evaluate for HTN. Women with rapid weight gain, edema, headache, blurred vision, epigastric pain, and unexplained nausea or vomiting were referred to the perinatologist for further evaluation to detect any case of gestational HTN and PE.

### Ethical Considerations

All processes in the study were in concordance with the principles accepted by the Ethics Committee of the Ministry of Health, Treatment, and Medical Education of Iran and approved by the Ethics Committee of Shiraz University of Medical Sciences (ethics code: IR.SUMS.MED.REC.1397.422). Also, written informed consent forms were taken from all participants; the evaluation was done namelessly, and the findings were stated to the participants. The study protocol also followed the Declaration of Helsinki ethical guidelines 1975.

### Statistical Analysis

Median± Inter Quartile Range (IQR=Q3-Q1) was used to describe a quantitative variable, and frequency (proportional frequency) was used to describe a qualitative variable. Kolmogorov-Smirnov, Mann-Whitney U, Wilcoxon signed-rank, logistic regression, and receiver operating characteristic (ROC) process with Uden index (sensitivity+specificity-1, which captures the performance of a diagnostic test in ROC) were used to determine the cut-off points. In addition, the positive predictive value (PPV) and the negative predictive value (NPV) were calculated using SPSS software version 22 (IBM, Armonk, New York, USA). A P=0.05 was considered as statistical significant level.

## Results

**Table:** Table1. Maternal Characteristics

**Features**		**Total (n=486)**	**Normotensive (n=449)**	**HPDs (n=37)**	**Gestational HTN (n=17)**	**PE**	
						**Mild (n=12)**	**Severe (n=8)**
	16-24	126(25.9)	108(24.1)	18(48.6)	9(52.9)	7(58.3)	2(25)
**Age (year), n (%)**	25-34	261(53.7)	251(55.9)	10(27)	5(29.5)	3(25)	2(25)
	≥35	99(20.4)	90(20)	9(24.4)	3(17.6)	2(16.7)	4(50)
	≤18.5	10(2)	9(2)	1(2.7)	1(5.9)	0(0)	0(0)
**BMI (kg/m^2^), n (%) **	18.5-24.9	397(81.7)	390(86.9)	7(18.9)	4(23.5)	2(16.7)	1(12.5)
	≥25	79(16.3)	50(11.1)	29(78.4)	12(70.6)	10(83.3)	7(87.5)
	1	164(33.9)	151(33.8)	13(35.2)	2(11.8)	5(41.7)	6(75)
	2	152(31.4)	135(30.2)	17(45.9)	11(64.7)	5(41.7)	1(12.5)
**Gravida, n (%)**	3	87(18)	82(17.9)	7(18.9)	4(23.5)	2(16.6)	1(12.5)
	4	46(9.5)	46(10.3)	0(0)	0(0)	0(0)	0(0)
	≥5	35(7.2)	35(7.8)	0(0)	0(0)	0(0)	0(0)

**HTN:** Hypertension; **PE:** Preeclampsia; **BMI:** Body mass index

**Table:** Table2. Lipid Profile of Studied Women in the First and Early Third Trimesters. Data Are Presented AS Median±IQR

**Parameters**	**Total**	**Normotensive**	**HPDs**	**Gestational HTN**		**PE**	
						**Total**	**Mild**	**Severe**
	TG	155±29	154±27	201±65	209±65	192.5±41	202.5±46.3	186.5±75
	TC	166.5±36	164±31	230±39	243±51	230±4	227.5±36.3	233±78.5
**First trimester**	HDL	43±14	43±14	42±8.5	39±9	42±6	42±11	43±6.2
	LDL	115±4	101±32	179±48	179±79	180±42.8	180.5±6	180±32
	LDL/HDL ratio	2.78±2	2.71±1.04	4.26±1.68	4.54±1.46	4.31±1.05	4.42±2.41	4.51±1.8
	TG	*188±29	*186±26	*248±2	*252±28	*245±13	*244.5±24	*247±15
	TC	*179±45	*175±41	*262±29	*261±42	*263±3	*249±33	*274±25
**Early third trimester**	HDL	*47±14	*46±15	*52±1	*52±1	*52±9	*50±10.6	*52±10.5
	LDL	*123±33	*121±28	*202±84.5	*200±7	*202±56	*201±88.8	*211±42.2
	LDL/HDL ratio	2.72±1	2.68±1.1	*3.88±2.1	*3.57±2.56	4.08±1.97	4.42±2.49	4.15±0.7

Values are measured as mg/dl. ^*^Significant difference with the first trimester **IQR:** Inter quartile range; **HTN:** Hypertension; **PE:** Preeclampsia; **TG:** Triglycerides; **TC:** Total cholesterol; **HDL:** High-density lipoprotein-cholesterol; **LDL:** Low-density lipoprotein-cholesterol;

**Table:** Table3. Comparison of Lipid Profiles among Studied Women in the First and Early Third Trimesters.

**Trimester**		**Normotensive vs. Gestational HTN **		**Normotensive vs. PE**		**Gestational HTN vs. PE**		**Mild PE vs. Severe PE**	
		**Mean rank**	**P-value**	**Mean rank**	**P-value**	**Mean rank**	**P-value**	**Mean rank**	**P-value**
	TG	169.18	<0.001	152.11	<0.001	-3.92	0.272	-3.92	0.263
	TC	231.45	<0.001	202.8	<0.001	-5.82	0.103	-5.82	0.589
**First trimester**	HDL	-42.76	0.241	-13.58	0.68	3.96	0.278	3.78	0.614
	LDL	194.88	<0.001	201.86	<0.001	1.04	0.772	1.04	0.938
	LDL/HDL ratio	178.69	<0.001	184.94	<0.001	-0.18	0.784	-0.98	0.936
	TG	252.18	<0.001	206.16	<0.001	-5.27	0.139	-5.27	0.616
	TC	223.56	<0.001	219.25	<0.001	-0.11	0.976	-0.11	0.031
**Early third trimester**	HDL	94.21	0.007	53.79	0.049	-3.21	0.367	-3.27	0.353
	LDL	208.16	<0.001	224.38	<0.001	1.58	0.604	1.85	0.562
	LDL/HDL ratio	130.72	<0.001	192.76	<0.001	3.37	0.345	3.37	0.877

**HTN:** Hypertension; **PE:** Preeclampsia; **TG:** Triglycerides; **TC:** Total cholesterol; **HDL:** High-density lipoprotein-cholesterol; **LDL:** Low-density lipoprotein-cholesterol;

Of 486 participants, 449 and 37 were normotensive and HPDs, respectively. Also, from 37 HPDs, 17 and 20 were gestational HTN and PE; and from 20 PE, 12 and 8 were mild and severe, respectively. More maternal age and higher body mass index (BMI) were seen in the PE group in comparison to the normotensive group (P=0.03 and P<0.001, respectively), but no difference was observed regarding gravida (P=0.3). Advanced age, high BMI, and higher gravida were seen in the gestational HTN group in comparison to the normotensive group (P=0.03, P=0.017, and P<0.001, respectively). However, there were no differences between gestational HTN and PE groups regarding maternal age, BMI, and gravida (P=0.3, P=0.2, and P=0.4, respectively); it was the same regarding the mild and severe PE groups (P=0.23, P=0.26, and P=0.45, respectively).

Maternal characteristics of 486 pregnant women are presented in Table-1. Thirty-seven (7.6%) patients developed HPDs during pregnancy, and 449 (92.4%) remained normotensive. Also, no case of eclampsia was observed. There are higher TG, TC, HDL, and LDL values in the early third trimester compared to the first trimester; however, no differences were seen in LDL/HDL ratio (Table-2). The LDL/HDL ratio was significantly lower in HDPs and gestational HTN (P=0.04 and P=0.02, respectively). In addition, TG had the highest increase among lipid profiles compared to the other values.

Lipid profile comparisons among normotensive, gestational HTN, PE, mild PE, and severe PE groups by the first and early third trimesters are shown in Table-3.

There were no differences in lipid profiles of TG, TC, LDL, HDL, and LDL/HDL ratio between the gestational HTN and PE group (Table-3). Also, no differences were observed between mild PE and severe PE groups (P>0.05, Table-3). TG, TC, LDL, and LDL/HDL ratios were higher among gestational HTN and PE groups compared to normotensive women (P<0.05 for all) by the first and early third pregnancy trimesters. Although the HDL level was higher among gestational HTN and PE compared with the normotensive group in the early third trimester (P=0.007 and P=0.049), no differences were seen in the HDL level in the first trimester (P>0.05, Table-3).

After adjusting for maternal age, BMI, and gravida, the values of TG, TC, LDL, and LDL/HDL ratio were significantly (P<0.001) associated with a higher risk of gestational HTN in the first (OR=1.03, 1.076, 1.049, and 3.684, respectively) and early third trimesters (OR=1.03, 1.078, 1.05, and 3.46, respectively). In addition, after adjusting for maternal age and BMI, the values of TG, TC, LDL, and LDL/HDL ratio were associated with a higher risk of PE in the first (OR=1.025, 1.035, 1.03, and 2.08, respectively) and early third trimesters (OR=1.026, 1.044, 1.03, and 2.14, respectively). However, adjusted logistic regression revealed no differences in terms of HDL levels in gestational HTN and PE groups compared with the normotensive group in the first trimester (OR=0.983 and 1.001) and in the early third trimester (OR=0.957 and 1.063).

The cut-off points of TG, TC, HDL, LDL, and LDL/HDL by the first and early third trimesters were estimated regarding gestational HTN and PE in Table-4. PPV and NPV were reported as well.

**Figure-1:**
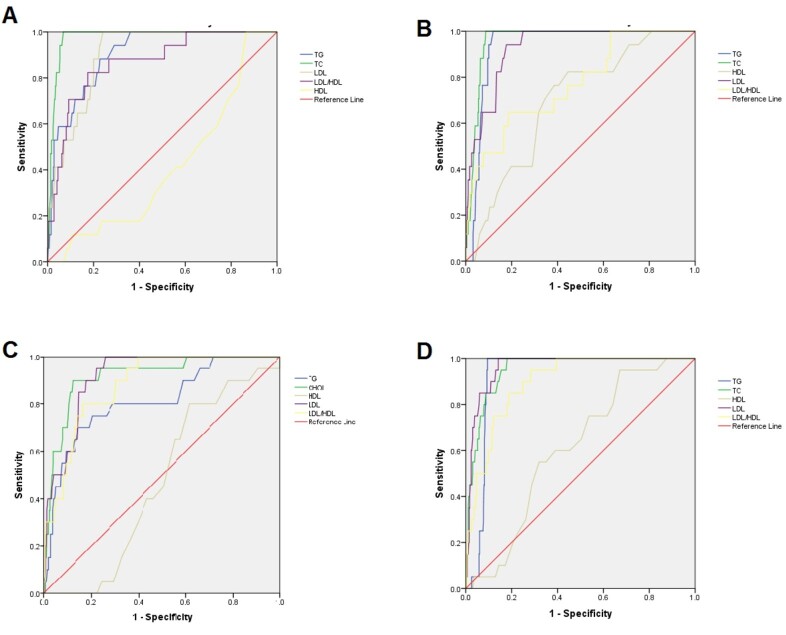
Figure 1. The receiver operating characteristic curves of lipid profiles among women with gestational HTN in the first (A) and third trimester (B) and women with preeclampsia in the first (C) and third trimester (D).

ROC curves of TG, TC, LDL, HDL, and LDL/HDL ratio cut-off points for gestational HTN and PE by the first and early third trimesters are presented in Figure-1.

## Discussion

**Table:** Table4. Cut-Off Point Values for the Lipid Profiles Based on Gestational HTN and Preeclampsia in the First and Early Third Trimesters

	**Trimesters**		**Cut-off point (mg/dl)**	**AUR**	**Sensitivity (%)**	**Specificity (%)**	**PPV (%)**	**NPV (%)**
		Gestational HTN	169.5	0.869	81.1	79.5	92.2	68
	TG	PE	180.5	0.813	70	85.6	80.5	77.6
		Mild PE	172.5	0.873	83.3	78.9	92.2	61.4
		Severe PE	180.5	0.707	62.5	84.1	92	42.7
		Gestational HTN	197.5	0.963	94.6	91.3	97	84.9
	TC	PE	197.5	0.917	90	88	86.4	91.4
		Mild PE	200.5	0.9	91.7	88.5	95.8	77.8
**First trimester**		Severe PE	184.5	0.921	100	74.5	92.1	100
		Gestational HTN	145.5	0.925	89.2	82	92.5	100
	LDL	PE	136	0.915	100	74.2	76.7	100
		Mild PE	136	0.9	100	73	91.7	100
		Severe PE	151.5	0.92	100	80.5	93.9	100
		Gestational HTN	3.616	0.686	82.4	82.5	93.3	60.9
	LDL/HDL ratio	PE	3.66	0.89	87.5	82	93.5	58
		Mild PE	3.41	0.869	64.7	81.4	93.3	60.9
		Severe PE	3.83	0.878	75	86.5	60.9	53.5
		Gestational HTN	217.5	0.904	100	92	97.3	19.6
	TG	PE	220	0.924	100	90.6	89.6	100
		Mild PE	220	0.916	100	89	96.4	100
		Severe PE	232	0.915	100	89.5	77	100
		Gestational HTN	234	0.974	91.9	94.7	98	91
	TC	PE	204	0.951	100	82	82	100
		Mild PE	210	0.928	100	83	94.7	100
**Early third trimester**		Severe PE	238.8	0.962	87.5	95	73.4	71.6
		Gestational HTN	151.5	0.965	97.3	86	95.4	91
	LDL	PE	155.5	0.962	100	85.5	85.2	100
		Mild PE	155.5	0.942	100	84.4	95	100
		Severe PE	185.5	0.968	100	98.4	75.9	100
		Gestational HTN	3.41	0.769	64.7	81.4	90.9	43.4
	LDL/HDL ratio	PE	3.97	0.852	100	80.8	91.2	82.6
		Mild PE	3.41	0.88	64.7	81.4	91.2	43.4
		Severe PE	3.71	0.902	91.7	70.5	90.3	73.8

**HTN:** Hypertension; **PE:** Preeclampsia; **TG:** Triglycerides; **TC:** Total cholesterol; **HDL:** High-density lipoprotein-cholesterol; **LDL:** Low-density lipoprotein-cholesterol; **AUR:** Area under ROC curve; **PPV:** Positive predictive value;** NPV:** Negative predictive value

In this study, 486 pregnant women were followed from the first trimester onward; 7.6% suffered from HPDs, of whom 3.5% had gestational HTN, and 4.1% had PE. In the PE group, there were significantly higher TG, TC, HDL, and LDL in the early third trimester compared to the first trimester. Maternal age and BMI were meaningfully higher in the PE group in comparison to the normotensive group; however, there was no significant difference in terms of gravida.

Age, BMI, and gravida were meaningfully higher in the gestational HTN group compared to the normotensive group, but no significant differences were seen among the gestational HTN and PE groups regarding maternal age, gravida, and BMI.

There were no meaningful differences in the lipid profiles between gestational HTN and PE groups. Also, no significant differences were seen between the mild and severe PE groups. While lipid profiles, age, and BMI were significantly higher in the gestational HTN and PE groups compared to the normotensive group in both the first and early third trimesters. Our findings regarding lipid profiles are almost in agreement with the related global statistics [2]

Increasing maternal age is reported to raise the chance of PE. 33.9% of the participants were experiencing their first pregnancies, from whom 55% and 75% developed PE and severe PE, respectively, considering first parity as a risk factor of gestational HTN [17].

There was a higher BMI in both gestational HTN and PE groups than in the normotensive group; however, they were the same among hypertensive and PE groups. These results agreed with the earlier studies considering BMI as a risk factor for PE [17][18][19].

Compared to the first trimester, almost all the lipid profile levels increased in the early third trimester; also, the result of the current study indicated that the maternal lipid levels of TG, TC, and LDL were accurate predictors of PE in the first and early third trimesters of that is consistent with the previous findings [11][15][20][21].

Although TG was a risk factor for PE in both the first and early third trimesters, previous research reported this correlation only in the early third trimester [22]. Furthermore, in other studies, the association between TG and mild/severe PE in the first and early third pregnancies was similar to our study with no significant statistical difference [23].

Considering the positive association of TG with gestational HTN, the results of the current study were similar to some of the previous studies [24][25]. However, no association was found between TG and gestational HTN [26]; the difference could be attributed to the difference in the blood sampling method. Also, in contrast to our study, they did not use fasting blood samples to acquire lipid profiles.

In the current prospective cohort study measuring the lipid profiles before the occurrence of PE in all participants, the causal relationship between the PE and lipid profile was demonstrated; however, they could not determine the severity of the diseases. In agreement with the current results, some cross-sectional studies measuring lipid profiles in pregnant women suffering from PE showed greater lipid profile levels in the PE group in comparison to the normotensive group [27][28]. Another study presented the change in TG, TC, LDL, and LDL/HDL ratio levels in the early second trimester with similar results to our study [29].

In another study, lipid profile levels, as well as the beta-hCG level of 184 pregnant women, were investigated at 14-18 weeks and 24-28 weeks. TC, TG, LDL, and beta-hCG were higher in the HPDs group than normotensive group regarding both GA periods [24], although some studies resulted in no correlation among the HDL level with HPDs, PE, and severe PE in the second trimester [15][21], some other studies showed that lower HDL level increased the risk of HPDs [20][30].

According to the current study, the most valid cut-off points regarding HPDs are LDL, TC, and TG in the early third trimester; TC and LDL in the first trimester. In Jin et al. study, the validity of TG was lower than the current study (AUC=0.736), and the sensitivity and specificity were reported as 85% and 64.8%, respectively [20].

The key points of the current study were the generalizability of the results due to the large sample size and the prospective design of the study. Also, lipid profiles among normotensive and HPDs subgroups, including gestational HTN and PE, were comprehensively investigated in the first and early third trimesters. Furthermore, the prevalence of HPDs and PE were estimated, and a variety of valid cut-off points were provided in the first and early third trimesters of a normal population in the south of Iran. The most important limitation of the current study was the absence of genetic and epigenetic factors associated with HPDs.

## Conclusion

Maternal lipid profiles are considered valid predictors of PE in the first and early third trimesters of pregnancy. These tests are available and inexpensive; also, by using the cut-off points, we could determine the high-risk pregnant women before developing PE and, consequently, other adverse pregnancy outcomes. Future studies, especially clinical trials, are recommended to investigate the efficacy of lipid-lowering drugs on PE patients.

## Conflict of Interest

The authors declare that there is no conflict of interest regarding the publication of this paper.
